# Impedimetric Bacterial Detection Using Random Antimicrobial Peptide Mixtures

**DOI:** 10.3390/s23020561

**Published:** 2023-01-04

**Authors:** Tal Stern Bauer, Ravit Yakobi, Mattan Hurevich, Shlomo Yitzchaik, Zvi Hayouka

**Affiliations:** 1 Institute of Biochemistry, Food Science and Nutrition, Robert H. Smith Faculty of Agriculture, Food and Environment, The Hebrew University of Jerusalem, Rehovot 76100, Israel; 2 Institute of Chemistry and the Center for Nanoscience and Nanotechnology, The Hebrew University of Jerusalem, Jerusalem 91904, Israel

**Keywords:** bacteria, random peptide mixtures, antimicrobial peptides, electrochemical impedance spectroscopy, self-assembly monolayer

## Abstract

The biosensing of bacterial pathogens is of a high priority. Electrochemical biosensors are an important future tool for rapid bacteria detection. A monolayer of bacterial-binding peptides can serve as a recognition layer in such detection devices. Here, we explore the potential of random peptide mixtures (RPMs) composed of phenylalanine and lysine in random sequences and of controlled length, to form a monolayer that can be utilized for sensing. RPMs were found to assemble in a thin and diluted layer that attracts various bacteria. Faradaic electrochemical impedance spectroscopy was used with modified gold electrodes to measure the charge-transfer resistance (R_CT_) caused due to the binding of bacteria to RPMs. *Pseudomonas aeruginosa* was found to cause the most prominent increase in R_CT_ compared to other model bacteria. We show that the combination of highly accessible antimicrobial RPMs and electrochemical analysis can be used to generate a new promising line of bacterial biosensors.

## 1. Introduction

Bacterial infections pose a public health crisis worldwide [1]. A crucial step in detecting the pathogenic status of a patient, as well as of food, surfaces, and medicinal equipment, is the identification of the bacterial infection source [2]. There is an urgent need for the development of rapid bacterial identification methods to improve treatment and minimize contamination and resistance-occurrence risks [3].

Electrochemical biosensors are integrated devices that convert specific biological interactions into electrical output that provides analytical information and are, hence, suitable for the detection of bacteria [4]. They can be fabricated at a relatively low cost and can provide a rapid and accurate analysis. Electrochemical impedance spectroscopy (EIS) is a powerful technique used for the analysis of the interfacial properties related to the bio-recognition events occurring at the electrode surface. Faradaic impedance measurement is performed in the presence of redox species. The changes in the charge-transfer resistance (R_CT_) translate the molecular-recognition event on the monolayer to a measurable signal [5]. Thus, EIS enables the label-free detection of biomolecules and biochemical interactions.

**Figure 1:**
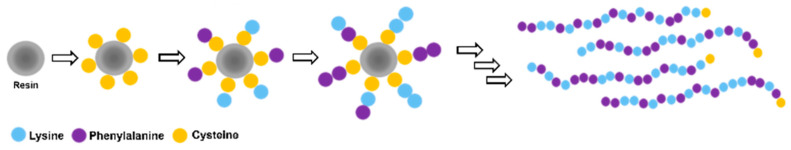
Solid-phase peptide synthesis of phenylalanine–lysine random peptide mixtures with cysteine at the C’ terminus (first coupling step: yellow circle). After the first coupling of cysteine, a 1:1 mixture of Fmoc–phenylalanine and Fmoc–lysine-(Boc) was coupled at each of the following 20 coupling steps to generate a 21-mer of Cys-[(Phe)_0.4_-(Lys)_0.6_]_n=20_, abbreviated as CFK.

Monolayers composed of aptamers, antibodies, polymers, and peptides are used for bacterial detection [4,5,6]. Among the variety of recognition molecules, peptides have great potential for being utilized in biosensing. Peptides, which are short chains of amino acids, are biocompatible and possess stability and selectivity by adopting a proper conformation toward the analyte. They are easily obtained and modified by chemical methods, allowing them to be tailored for specific surface chemistry applications [7]. Thiol–Au is the simplest and most common strategy for assembling peptides monolayers [8,9]. The process of self-assembled monolayer (SAM) organization includes an initial stage of fast S-Au bond formation. The second stage is a slow organization process where the immobilized molecules assemble to maximize their intermolecular interactions [9,10]. While interactions between the peptides might stabilize the monolayer and lead to a high density, repulsion due to electrostatic and/or steric hindrance can hinder monolayer assembly [11]. Antimicrobial peptides (AMPs) are short natural or synthetic peptides that eradicate bacteria through interactions with their membrane. While there is a high diversity in the sequences, lengths, and structures of AMPs, the different variants have two features in common: cationic charges and a high degree of hydrophobicity. Random peptide mixtures (RPMs) are peptides that optimize the advantage of AMPs’ common features—their 20 amino acids chain length combines hydrophobic and cationic amino acid residues in random sequences [12]. RPMs are easy to synthesize in large quantities (Figure 1), and an analysis showed that their synthesis is reproducible [13]. When equal ratios were used for coupling during the synthesis, this resulted in a ratio close to 1:1, in line with the features of the amino acids used with a minor favor towards hydrophobic amino acids [12]. Our previous studies revealed that RPMs have broad-spectrum antimicrobial activity [12,13,14,15,16,17]. This activity was preserved at varying pH values and physiological media, as well as in mice models with high efficacy and safety [18,19,20]. Of the multiple possible combinations of hydrophobic and cationic amino acids, those containing phenylalanine and lysine were among the combinations with the highest antimicrobial activity. Furthermore, we explored their possible mechanism of action and found that it involves electrostatic and hydrophobic interactions with the negatively charged bacterial membrane, via either pore-formation or non-selective distribution on the membrane [14]. Lastly, RPMs immobilized on polystyrene beads bind and eliminate bacterial cells very efficiently [18]. Since RPMs have a demonstrated ability to bind bacteria based on their intrinsic features of hydrophobicity and a positive charge, they have the potential to be used as recognition molecules for the electrochemical sensing of bacteria, with the considerable advantage of eliminating the need to synthesize a peptide with a specific sequence, which is time-consuming and expensive. The straight-forward and cost-effective production process, which does not involve tedious purification protocols, using RPMs as recognition molecules has many advantages compared to using standard peptides.

**Figure 2:**
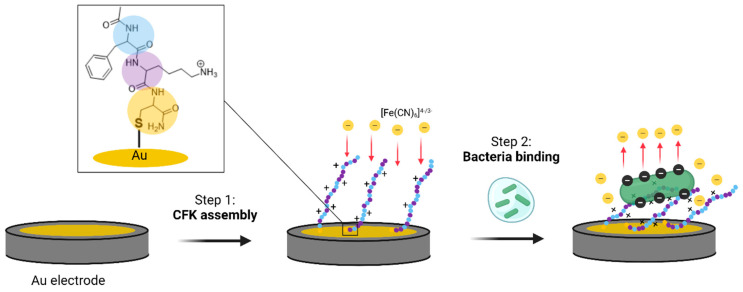
A graphical presentation of the study: CFK assembly on Au surfaces (step 1) and its rearrangement after bacterial binding (step 2). The charge-transfer resistance (R_CT_) increases in response to the bacteria adhesion due to its electrostatic repulsion of the redox active species [Fe(CN)_6_]^3−/4−^.

However, unlike the assembly of homogeneous peptides on gold, which is expected to self-organize and to form strong intermolecular interactions, the assembly of RPMs has not been explored yet. RPMs contain mixtures of entities with different sequences and a distribution of positive charge/hydrophobic residues along the chain length that may affect the assembly. Here, we studied the ability of RPMs to form a monolayer on a gold surface and their potential use for bacterial sensing using EIS (Figure 2).

## 2. Experimental Section

### 2.1. Materials

All solutions were prepared with triple-deionized water (TDW, 18.3 MΩ∙cm, Millipore Milli-Q, Bedford, MA, USA). The buffer solution was PBS in low ionic strength (PBS-L) containing 10 mM Na_2_HPO_4_·7H_2_O, 1.8 mM KH_2_PO_4_, 1.37 mM NaCl, and 0.027 mM KCl (pH 7.4). Electrochemical analyses were conducted with Metrohm-Autolab PGSTAT-12 digital potentiostat (EcoChemie BV, Utrecht, The Netherlands), operated with Nova software for utilizing electrochemical impedance spectroscopy (EIS). A three-electrodes cell was used for the measurements: Ag/AgCl (in 3 M KCl) as reference electrode (RE) and a Pt wire as a counter electrode (CE). Polycrystalline bulk gold electrodes with a 2 mm diameter were used as working electrodes (WE) (CH instruments, USA). WE were manually polished on micro-cloth pads (Buehler, Lake Bluff, IL, USA) with deagglomerated 0.05 μm alumina suspension (Buehler). After polishing, the electrodes were washed with TDW. Bacterial strains used in the research are *E. coli* K12 MG1655 (rp), *Pseudomonas aeruginosa* PAO1, *Staphylococcus aureus* Newmann (kindly received from Prof. Roni Shapira’s lab, HUJI), Methicillin-resistant Staphylococcus aureus (MRSA), *Bacillus subtilis* 168, *E. coli* K12 BW25113 (kindly received from Prof. Shimshon Belkin’s lab, HUJI). All bacteria were grown in Luria broth (LB) at 37 °C.

### 2.2. Methods

#### 2.2.1. Synthesis of CFK Random Peptide Mixtures (RPMs)

CFK random peptide mixtures were synthesized using standard Fmoc-based solid-phase peptide synthesis (SPPS) with a Liberty Blue™ automated microwave peptide synthesizer (CEM Corp., Matthews, NC, USA) as was published previously [12]. Cys-[(Phe)_0.4_-(Lys)_0.6_]_n=20_ (abbrev. CFK) was synthetized on 0.25 mmol scale on rink amide resin (loading 0.53 mmol/g (ChemImpex, IL, USA)) as the solid support. After swelling (5 min) of the resin, Fmoc removal (deprotection) was completed with 20% *v*/*v* piperidine/DMF. Standard couplings of amino acids were carried out at 0.1 M in N,N-Dimethylformamide (DMF) using DIC/OxymaPure^®^ activation (0.2 and 0.1 M, respectively). Fmoc–Cysteine–Trt was coupled first at 50 °C, followed by deprotection. The next 20 coupling steps were carried out by using 1:1 molar ratio mixture of protected α-amino acids: Fmoc-L-Phenylalanine with Fmoc-L-Lysine-Boc. At the end of the synthesis, the resin was washed with DMF and DCM and dried with methanol and diethyl ether. The peptides were cleaved from the resin by mixing the resin with a 4 mL cleavage cocktail of 92.5% Trifluoroacetic acid (TFA), 2.5% 1,2-Ethanedithiol, 2.5% triisopropylsilane (TIPS), and 2.5% TDW for 3 h with agitation. After filtration, the peptides were precipitated from the TFA solution by the addition of cold diethyl ether and collected by centrifugation (Eppendorf R5810 8000 rpm for 10 min). The ether was then removed, and the process repeated once more. The peptides were dried under a stream of nitrogen, dissolved in 20% ACN/TDW, and lyophilized. The product was analyzed by MALDI TOF/TOF AutoFlex Speed (Bruker Daltonics Inc., Bremen, Germany) to evaluate the success of synthesis.

#### 2.2.2. CFK Assembly on a Gold Surface

Gold substrates for surface characterization were prepared by evaporation of 10 nm chrome as adhesion layer followed by evaporation of 100 nm Au on top of highly doped n-type Silicon wafer. The surfaces were washed with ethanol, dried under a mild stream of nitrogen gas, and cleaned using UVOCS (ultraviolet ozone cleaning system) for 20 min.

Solution of 1.4 mg/mL (0.5 mM) CFK was prepared in PBS-L buffer solution (pH = 7.4). A variety of buffers was examined. Finally, the adsorption process was completed in PBS buffer with low ionic strength (PBS-L) in order to enable the optimal organization and interaction of the peptides chains. Chemisorption was performed by drop casting CFK solution on bare gold substrates for an overnight incubation (16 h) at 37 °C. The surfaces were washed after incubation with PBS-L buffer and TDW and dried under nitrogen stream. Characterization of Au-CFK surfaces is described at the next section.

#### 2.2.3. Surface Characterization

Variable angle spectroscopic ellipsometry (VASE) measurements were carried with a VB-400 ellipsometer (J.A. Woollam Co., Inc., Lincoln, NE, USA). The optical thickness of the random peptides layer on the gold surface was measured by fitting the results to the CAUCHY model, when the values are an average with standard deviation of surfaces from different batches.

Kelvin-probe-derived contact potential difference (CPD) measurements were performed with a Kelvin probe S vibrating gold grid reference electrode (WF—4.8 eV), operated by the Kelvin control 07 unit (DeltaPhi Besocke, Julich, Germany), in a specially built Faraday cage under inert argon atmosphere. Ohmic back contacts were made with eutectic Ga-In (99.99%, Sigma-Aldrich, Darmstadt, Germany), and the CPD signal was recorded using Keithley 2450 SMU. The measurements were taken after the few minutes needed for the signal to stabilize and were performed with respect to a reference electrode.

X-ray photoelectron spectroscopy (XPS) spectra were recorded with a Kratos Axis Supra^+^ spectrometer (Kratos Analytical Ltd., Manchester, UK) using an Al K_α_ monochromatic radiation X-ray source (1486.7 eV). Data were collected and analyzed by a Casa XPS (Casa Software Ltd., Devon, UK) and Vision data-processing program (Kratos Analytical Ltd.). The high-resolution XPS spectra were collected with a take-off angle of 90° (normal to analyzer); vacuum conditions in the chamber were 1.9 nTorr, for the C 1s, O 1s, N 1s, S 2p, and Au 4f levels, with pass energy of 20 and 0.1 eV step size. The binding energies were calibrated using C 1s peak energy as 285.0 eV.

Reductive desorption analysis conducted by cyclic voltammetry (CV) was performed to determine the surface coverage of the peptides mixture, termed FK, on Au electrodes, by scanning over the potential range of −0.5 to −1.4 V at the scan rate of 150 mV/s in 0.1 M KOH solution. The electrolyte solution was degassed with nitrogen stream prior to CV scans.

Atomic force microscopy (AFM) analysis on ultra-flat gold substrates was performed using a Dimension Icon XR probe microscope (Bruker) in tapping mode. Ultra-flat gold substrates were prepared as previously described [21]. Au (100 nm) was evaporated on 3 × 3 cm^2^ pieces of highly doped n-type Silicon wafer. An optical adhesive (NOA 88, Norland products, NJ, USA) was used for attaching a glass slide to the surface of the Au. The OA was cured by exposing UV LED (λ_max_ = 365 nm) for 20 min. The glass/OA/Au composite was carefully separated from the silicon template to expose the smooth surface at the Au/silicon interface. The assembly of CFK was performed as described in Section 2.2.2.

#### 2.2.4. Florescence Microscopy

Fluorescence microscopy was used to evaluate the binding ability of bacteria to CFK-modified Au surfaces. The bacteria cells were grown overnight in LB at 37 °C. The bacterial cells’ suspension was diluted 1/50 in fresh LB and grown for additional 3–4 h. Bacteria pellets were washed twice with PBS-L by centrifugation (8000 rpm for 2 min). The bacteria pellet was diluted to OD_600 nm_ = 0.5 and stained with Syto9 (live, green; Life Technologies Corp., Carlsbad, CA, USA). The suspension was dropped on the immobilized peptide surfaces and incubated for 40 min at room temperature. The surfaces were washed with PBS-L and TDW (three times) to remove unbound bacteria and observed with a fully motorized IX81 fluorescent microscope (Olympus, Tokyo, Japan). Images were captured at ×20 magnification in at least three distinct areas of the sample. PMT emission was 490–530 nm.

#### 2.2.5. Gold Electrodes’ Surface Modifications and Impedimetric Sensing

WE were dipped in 0.5 mM (FK)_20_-Cys in PBS in low ionic strength (PBS-L) (pH 7.4) for 16 h at 37 °C, rinsed by dipping in PBS-L, and then measured. Subsequently, the electrodes were exposed to 10^8^ CFU/mL bacterial cells’ suspension in PBS-L for 40 min under gentle agitation. The bacteria cells were grown overnight in LB at 37 °C. The bacterial cells’ suspension was diluted 1/50 in fresh LB and grown for additional 3–4 h. Bacteria pellets were washed twice with PBS-L by centrifugation (8000 rpm for 2 min) and diluted to an optical density of 0.5 at 600 nm. Following incubation with bacteria, the electrodes were washed and measured. The EIS characterization was prepared in an EIS solution, containing 1 mM K_3_[Fe(CN)_6_] and 1 mM K_4_[Fe(CN)_6_] (RedOx species) in PBS-L. Spectra were recorded by applying a single sine AC potential of 10 mV amplitude superimposed with 0.21 V DC potential (vs. RE) and scanning over the frequency range of 100 kHz to 0.1 Hz. The data were analyzed as Nyquist plots and fitted to a Randles-like equivalent circuit of $R_{S}\left[(R_{CT}W\right)Q],\;$where R_S_ is the solution resistance, R_CT_ is the interface charge-transfer resistance, W is the Warburg diffusion element, and Q is the constant phase element of non-ideal capacitance. All experiments were completed on at least 3 samples with at least 3 repetitions.

## 3. Results and Discussion

RPMs were synthetized with a 1:1 mixture of phenylalanine and lysine in each coupling step of the solid-phase peptide synthesis. The product is a mixture of 2^n^ possible different sequences composed of only phenylalanine and lysine (Figure 1). The peptides mixture, termed FK, was previously shown to have significant antimicrobial activity against a broad spectrum of bacteria, by binding their membrane [13,14,15,16,17]. Here, we equipped the FK RPMs with a cysteine moiety at the C terminal to allow anchoring to gold surfaces, termed Cys-[(Phe)_0.4_-(Lys)_0.6_]_n=20_ (CFK). CFK was analyzed by MALDI in order to verify the length of peptides chains and the ratio between the amino acids. MALDI analysis revealed several peaks around 2.8 kDa, which correlates with a 21-mer peptide composed of one cysteine and a mixture of phenylalanine and lysine (Figure S1). The most common peptide combinations were found to be composed of sequences with a 3:2 F:K ratio. The higher content of hydrophobic amino acid observed for CFK can be attributed to the relative coupling reactivity of the two amino acids [12,13,14].

FK terminated with cysteine at the C-terminal (CFK) was incubated on gold electrodes or surfaces. The thiol chemisorbs onto the gold via the formation of a S–Au bond, and the peptides self-organized to form a monolayer [6]. The resulting layers could then be characterized through various methods for surface-chemistry analysis. To characterize the monolayer, CFKs were assembled on Au-coated Si wafers (**Au-CFK**). VASE analysis showed an additional thickness of 15.3 (±0.8) Å (MSE = 5.45). The deviation of the measured thickness from the theoretical length of the peptides (~47 Å) indicates that CFK forms a monolayer and that either the RPMs are tilted or the assembly did not reach full coverage. The surface number density (N_S_) of the CFK monolayer was extracted from reductive desorption analysis [22]. The concentration of CFK was calculated by the characteristic peak at −1.1 V (Figure S2), which yielded N_S_ = 1.4 × 10^−10^ mole CFK/cm^2^. A typical N_S_ of alkanethiol SAM is 9.3 × 10^−10^ mole RSH/cm^2^ [23]. Our results show that the CFK surface coverage is lower than that of typical alkanethiols. This may be due to repulsion between the peptide chains. Consistent with CFK RPMs being positively charged molecules with substantial intermolecular electrostatic repulsion, we found our N_S_ comparable to other charged alkanethiol monolayers [24]. Our reductive desorption studies support these VASE results, confirming that CFK assembly results in about 10% coverage.

**Figure 3:**
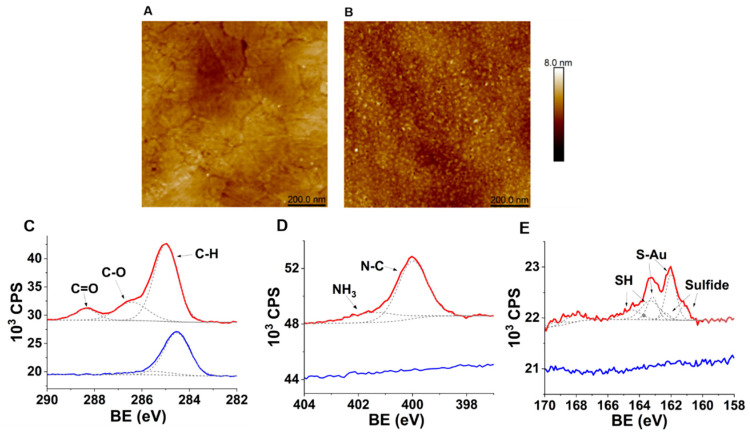
Characterization of **Au-CFK** surfaces. **Upper panel:** AFM analysis of the modified gold substrates. (**A**) Bare gold (Ra = 0.26 nm); (**B**) after-adsorption CFK random peptide mixtures (Ra = 0.31 nm). **Lower panel:** XPS spectra of CFK-Au surface: (**C**) C 1s BE region; (**D**) N 1s BE region; (**E**) S 2p BE region (red lines) and bare Au surface (blue lines). Recorded data are shown with lines, and the Gaussian fit is shown with dashed lines.

AFM analysis was performed to evaluate the topography of the CFK peptide monolayer on the gold surface. The results show that while the average surface roughness (Ra) of bare gold is 0.26 nm, the FK monolayer assembly results in only a slight increase to 0.31 nm (Figure 3A,B). This moderate increase in roughness further hints at the assembly of a peptides monolayer on the surface [25].

The surface potential caused by adsorption of the CFK monolayer on Au was measured using CPD. The CFK assembly yields a ΔCPD of −0.94 (±0.08) V with respect to the bare gold substrate. The decrease in the surface potential indicates that the dipole is directed toward the surface. This proves that the surface is covered with positively charged molecules and confirms the presence of lysine-rich CFK peptides.

The assembly of CFK peptides on the gold surface was further characterized using XPS analysis, to trace the peptides at the atomic level features: sulfur, nitrogen, and carbon atoms. A binding energy (BE) peak that corresponds with the C=O bond of carbonyl in peptide chains was observed at 288.3 eV (Figure 3C) [26]. The two peaks at the nitrogen-related binding energy spectral region, 400 eV and 401.5 eV, correspond with the primary amine and the quaternary amine, respectively (Figure 3D) [27]. BE peaks at 162.1, and 163.2 eV corresponds with the 2p electrons of the sulfur attached to the gold (Figure 3E). The peaks at 163.4 and 164.6 eV are related to the 2p electrons of the unbound thiols (S-H) [28]. The ratio between the S-Au-related peaks and the SH ones (1:1.35) suggests that most of the CFK is chemisorbed, while part of it is physisorbed as π–π interactions between peptide chains, or that interactions of amine side chains with gold may be contributing to the monolayer [29]. Atomic percentages of Au, C, and N were 32%, 47%, and 7%, respectively. The C/Au ratio is 1.4, indicating that most of the Au surface is covered by the peptide monolayer.

**Figure 4:**
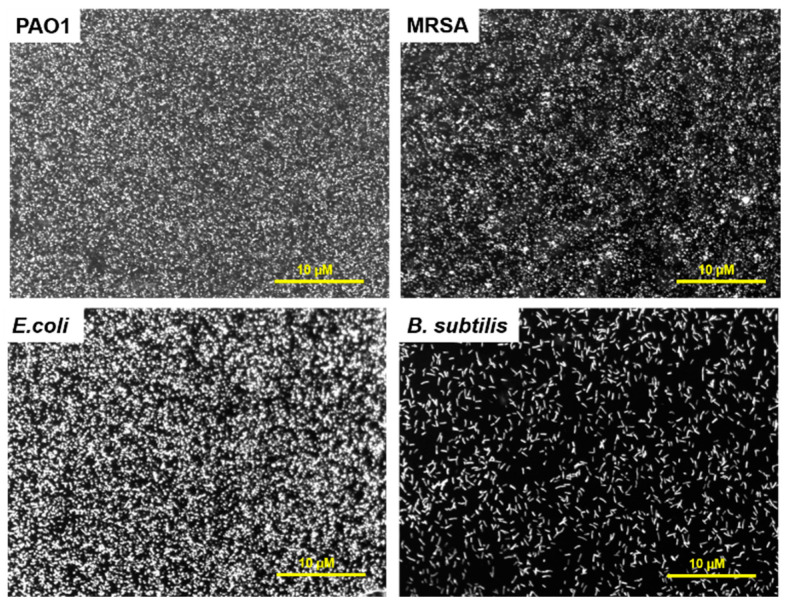
Binding of various bacterial cells to **Au-CFK**. Bacteria were stained with syto9 and incubated on FK-modified surface. The unbound bacteria were washed, and the surfaces were observed via a fluorescence microscope (×20 magnification).

The CFK monolayers binding with the bacteria were evaluated. The interaction of FK with bacteria in solution was demonstrated previously [13,14]; however, when peptides are anchored to a surface, their interaction with the bacteria might decrease, as their flexibility and their folding into secondary structures can be limited [18,30]. To study the ability of the CFK layer to attract bacterial cells, various Gram-positive and Gram-negative bacterial cells were stained with a syto9 fluorescent dye and incubated on the CFK-modified gold surfaces. Strains included *Pseudomonas aeruginosa* (PAO1), *E. coli*, methicillin-resistant *Staphylococcus aureus* (MRSA), and *Bacillus subtills*. The binding of all bacterial cells to **Au-CFK** surfaces was observed at different microbial loads, with maximal coverage gained at 10^8^ CFU/mL (Figure 4 and Figure S3). High coverage of the surface was obtained for PAO1, MRSA, and *E. coli*; less coverage was observed in *B. subtilis*. In a control experiment, the binding of bacteria to a bare Au surface was minor (Figure S4). **Au-CFK** analyses demonstrated that the anchored peptides maintain their bacterial-binding properties.

We next developed a sensing platform for detecting bacteria using electrochemical sensing. EIS was performed before and after adsorption of CFK to the electrodes, forming the peptide monolayer on the gold electrode (**AuE-CFK**). To quantify the impedimetric response, data were fit to a Randles circuit [31]. The resistance and capacitance components of the system were extracted. A change in the ratio between the resistance and the capacitance was observed, depending on the bacteria species. However, since the dominant change was in the resistance parameter, the response to the binding bacteria was analyzed according to the changes in the resistance for redox-active [Fe(CN)_6_]^3−^/[Fe(CN)_6_]^4−^ mobility. The charge-transfer resistance (R_CT_) values for **AuE-CFK** were in the range of 600–2000 Ω compared to 80 Ω for the bare Au electrode. Subsequently, EIS measurements of **AuE-CFK** were performed in response to incubation with different bacteria (at the same microbial load).

**Figure 5:**
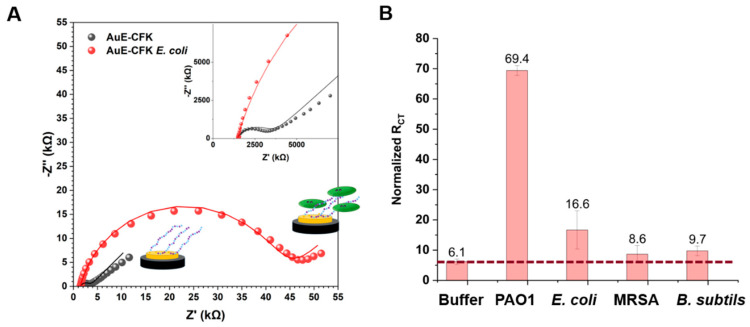
Impedimetric response of **AuE-CFK** to exposure to bacteria. (**A**) A Nyquist plot of **AuE-CFK** before (black) and after (red) a 40 min incubation with *E. coli*. Raw data are presented as circles and as lines when fit to a Randles circuit. (**B**) Normalized R_CT_ values of **AuE-CFK** after exposure to bacteria. Results are Avg + SD of 3 different electrodes with 3 exposure repetitions.

After incubation with bacteria or buffer (negative control), the electrodes were washed with buffer to remove unbound bacteria and measured again. The incubation with 10^8^ CFU/mL *E. coli* resulted in an R_CT_ increase of ~40,000 Ω (Figure 5A). **AuE-CFK** was incubated with three other bacteria (Figure 5B and Figure S5). The increase in R_CT_ was bacteria-dependent. PAO1 had the strongest response, showing a 69-fold increase. *E. coli* presented a 16-fold increase in R_CT_, while MRSA and *B. subtilis* both yielded a rather weak response. Incubation of the layer in buffer is expected to also cause an increase in R_CT_, due to the re-organization of the layer resulting in a change in the impedimetric signal [32,33]. Specifically, since the CFK monolayer is composed of different sequences, due to the intramolecular interactions between the positively charged lysine residues and the bulky phosphate anion of the buffer, the monolayer is densified and exposes a negatively charged interface that repels the negatively charged redox active couple, ferri-/ferro-cyanide. However, the response of CFK with PAO1 is an order of magnitude larger than the response of the buffer only, showing a specific and tangible detectability of the bacteria.

The binding of bacteria to CFK surfaces that was observed by fluorescent microscopy did not entirely translate to an EIS signal, which requires a deeper evaluation of the observed effect. Since the peptides have antimicrobial activity when in solution, we examined whether the signal related to the CFK surfaces was caused by either dead bacteria or the redox active species effect itself. Our control studies showed that dead bacterial cells do not lead to an EIS response and that the redox species do not affect the bacteria’s viability nor their removal from the surface (Figures S6–S8).

EIS response results from the permeation of the redox active species through the monolayer, which, hence, depends on the changes in the monolayer charge, dipole, density, and morphology; this might be affected by the nature of the analyte attached to the substrates. The CFK monolayer is positively charged and can interact with [Fe(CN)_6_]^3−/4−^ via electrostatic interactions. While bacteria are attached, the positive charge of CFK can be shielded, and the changes in the redox species permeation could result in an increase in R_CT_ (Figure 2). Gram-negative bacteria have more negatively charged surface density than Gram-positive bacteria [34]. Therefore, interactions of [Fe(CN)_6_]^3−/4−^ with PAO1 and *E. coli* may result in larger electrostatic repulsion in comparison to MRSA and *B. subtilis* and in a higher EIS response. The differences between the responses to PAO1 and *E. coli* are due to the nature of their membranes and of the other components on the membrane, such as receptors, saccharides, etc. It is known that the Zeta potentials of PAO1 and *E. coli* have different values [35]. The PAO membrane contains a few components that carry a dominant weight in its surface total charge. Although the main phospholipid of PAO and *E. coli* is phosphatidylethanolamine, which is zwitterionic [36], PAO has an unusually high phosphorous content, contributing to its overall negative surface charge [37]. The LPS of PAO contains special acidic saccharides such as 2-keto-3-deoxyoctulosonic acid (KDO), 2-amino-2-deoxyuronic acids, 2,3-diamino-2,3-dideoxyuronic acids, and 5,7-diamino-3,5,7,9-tetradeoxynonulosnic acids. The PAO capsule also consists of negatively charged o-capsular and alginate polysaccharides [37,38]. All those components may contribute to the negative charge of PAO and to the repulsion effect on the redox active species and, subsequently, provide the basis for the high EIS signal.

## 4. Conclusions

We demonstrate a new strategy for bacterial detection, which relies on readily accessible RPMs as recognition monolayers. Among antimicrobial peptides, the random peptide mixture, CFK was demonstrated to target a broad spectrum of bacteria. Here, for the first time, the co-assembly of random peptides was utilized to form the active layer of an electrochemical bacterial biosensor. We showed that those accessible RPMs can be assembled on a gold surface while maintaining bacteria-binding properties. In their immobilized state, CFK mixtures can bind various bacterial cells to the surface. The translation of the binding to an electrochemical signal was achieved by EIS methodology, resulting in a high signal for PAO1, due to its negatively charged extra-cellular components. This study proves that RPM-based biosensors can be used for bacteria detection. Their advantages from the applicability point of view are a straight-forward synthesis protocol that does not require purification, a low cost of production, stability in different environments, and an affinity to a wide range of bacteria. The different responses of **AuE-CFK** to the various bacteria species observed in this study require further analysis. Applying alternative electrochemical methods can be used to further utilize the AuE-CFK for bacteria biosensing. Further optimization of the device architecture can also be applied to increase the sensitivity, thus offering a new and acutely needed application for this very intriguing family of synthetic peptides.

## Data Availability

The data that support the findings of this study are available in the supplementary material of this article.

## Supplementary Materials

The following supporting information can be downloaded at: https://www.mdpi.com/article/10.3390/s23020561/s1.
