# Supplementary material for: Impedimetric Bacterial Detection Using Random Antimicrobial Peptide Mixtures
Source: Sensors (Basel). 2023 Jan 4;23(2):561. doi: 10.3390/s23020561 (PMC9866871; doi:10.3390/s23020561)
Supplement: Supplementary file 1 [file sensors-23-00561-s001.zip › sensors-2055068-supplementary.pdf]

## Supporting information

# Impedimetric Bacterial Detection Using Random Antimicrobial Peptide Mixtures

**Tal Stern Bauer [1,2], Ravit Yakobi [2], Mattan Hurevich [2], Shlomo Yitzchaik [2] and Zvi Hayouka [1,*]**

[1] Institute of Biochemistry, Food Science and Nutrition, Robert H. Smith Faculty of Agriculture, Food and Environment, The Hebrew University of Jerusalem, Rehovot 76100, Israel
[2] Institute of Chemistry and the Center for Nanoscience and Nanotechnology, The Hebrew University of Jerusalem, Jerusalem 91904, Israel
* Correspondence: zvi.hayouka@mail.huji.ac.il

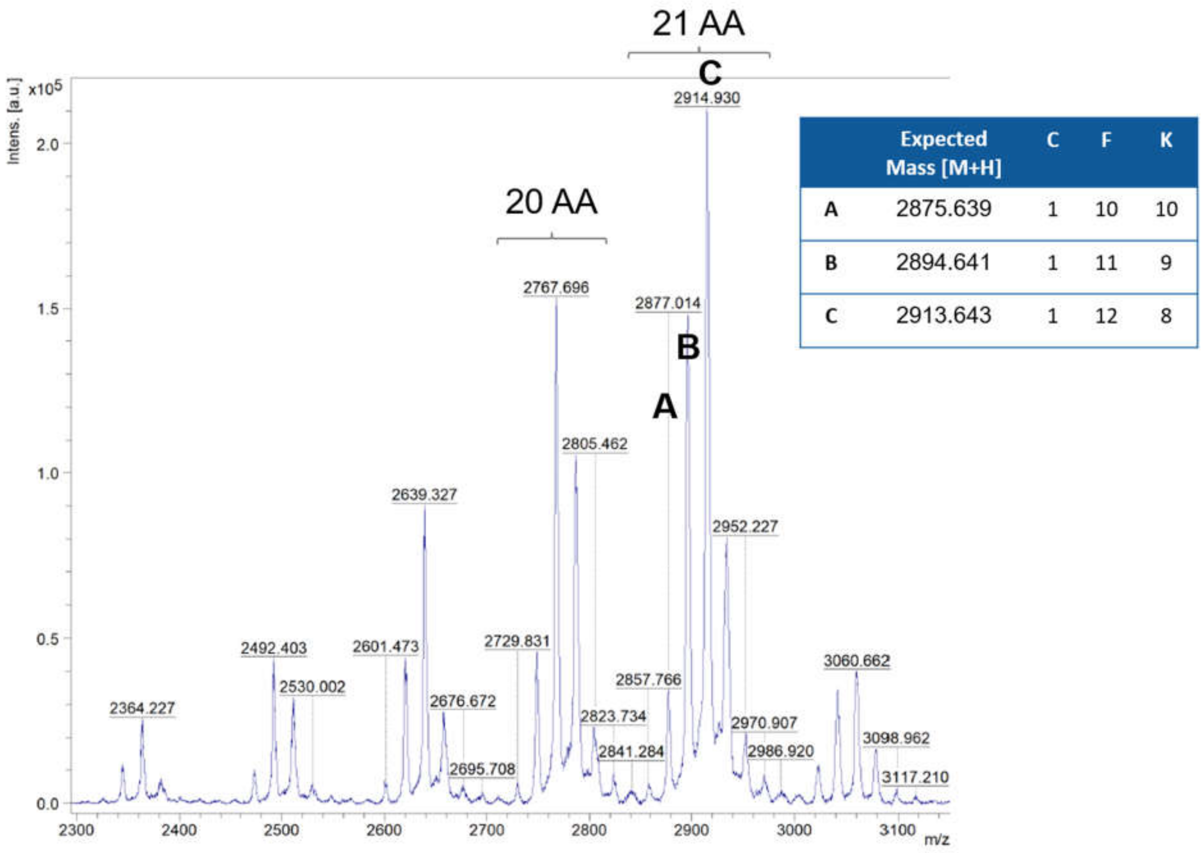


**Figure S1.** MALDI TOF/TOF analysis of CFK. Averaged expected molecular mass is 2875.639 Da. The main peak corresponds to 21-mer with distribution of F:K ratio.

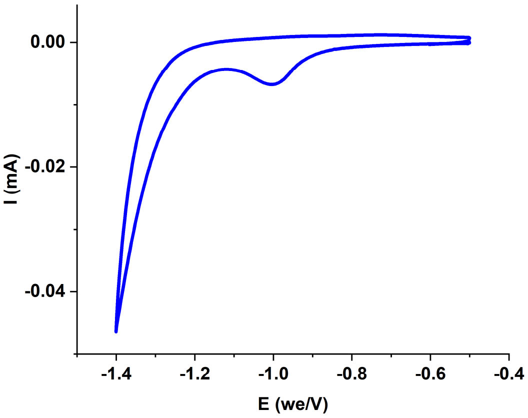


**Figure S2.** Reductive desorption of FKC modified Au electrode. The CV recorded in 0.1 M KOH solution from -0.5 to 1.4 V at the scan rate of 150 mV.

$$Au\text{-}SR + e\text{-} \rightarrow Au + SR^{-}$$

From the CV data, we were able to calculate the density of FK molecules according to equation:

$$\mathrm{FK} = \frac{\mathrm{Q} * \mathrm{C}}{\mathrm{A}}$$

Here, FK is the amount of FK molecules, Q is the charge transfer (calculated by the integral of the CV peak), C is the coulomb constant and A is the area of the electrode. We obtain FKC surface concertation of 2.5 molecules/nm$^2$ = 1.4*10$^{-10}$ moles/cm$^2$.

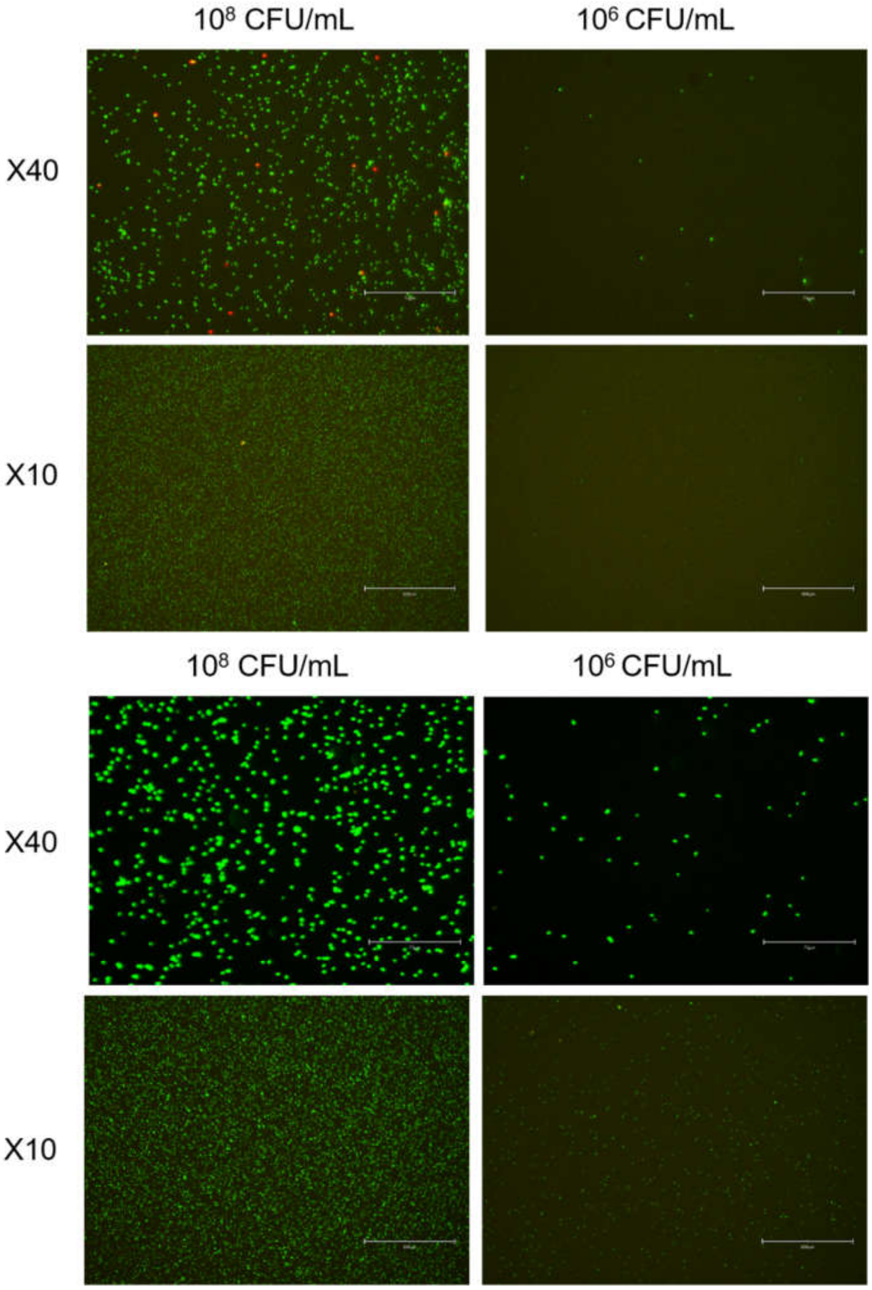


**Figure S3.** Coverage of Au-FKC surfaces with $10^8$ and $10^6$ CFU/mL MRSA and *E.coli* in 2 magnifications as observed by EVOS fluorescence microscopy. Scale bar: magnification X40 – 75µM, magnification X10 – 300 µM.

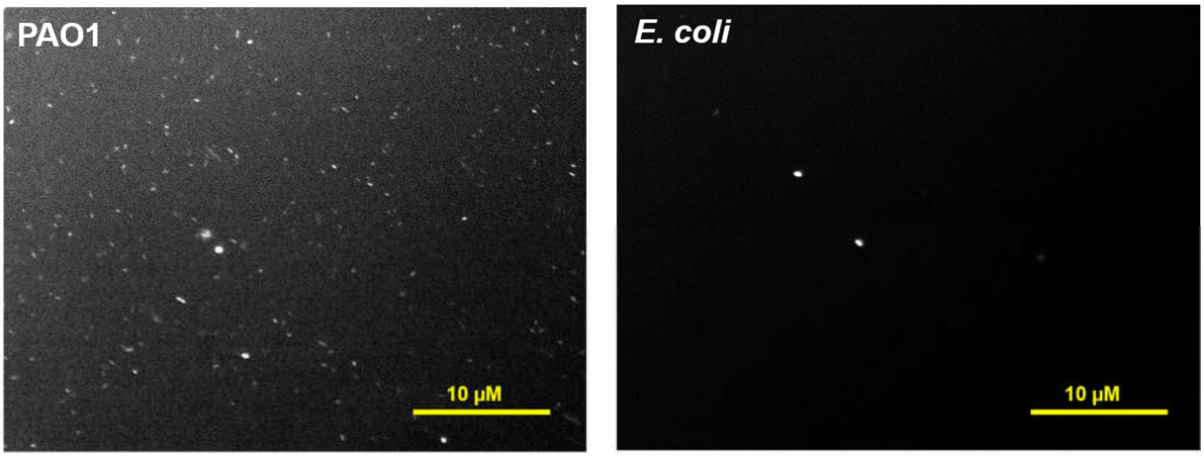


**Figure S4**. Binding of bacteria to bare Au as observed by fluorescence microscopy, magnification X20.

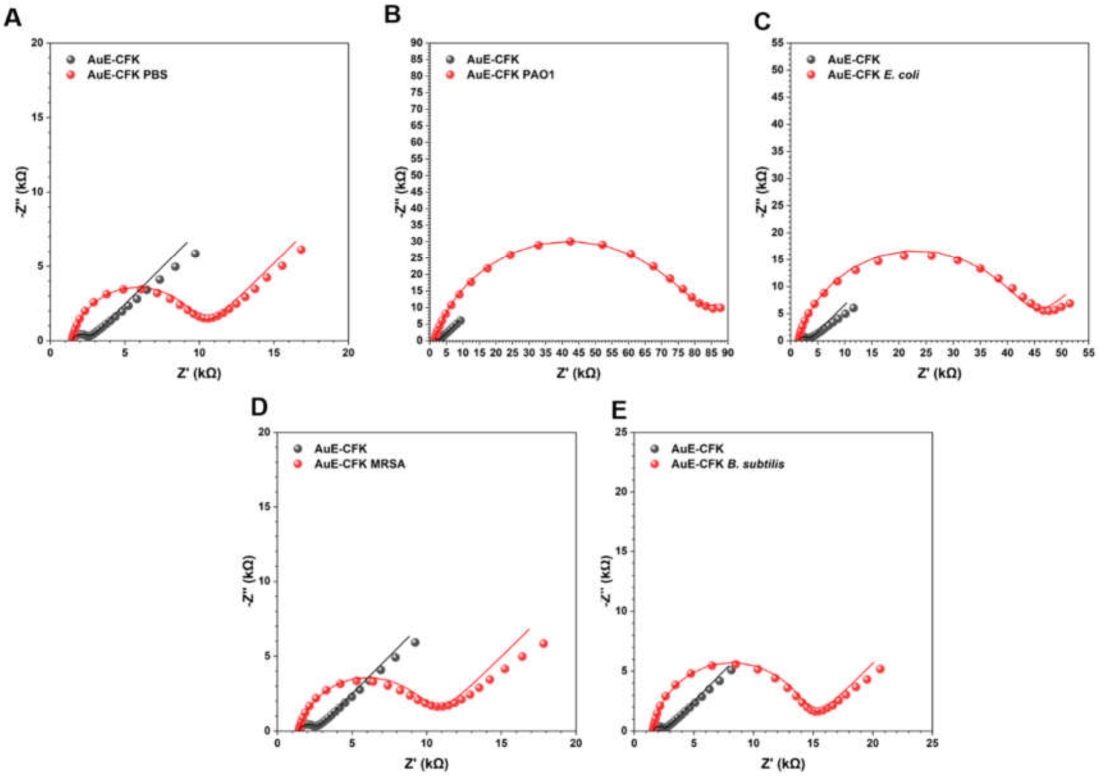


**Figure S5.** Nyquist plot of AuE-CFK before (Black) and after 40 min (Red) exposure to bacteria. Raw data are presented as circles, and as lines when fit to a Randles circuit. (**A**). PBS (**B**). PAO1 (**C**). *E. coli* (**D**). MRSA (**E**). *B. subtilis*. Results are Avg+SD of 3 experiments with 3 biological experiments.

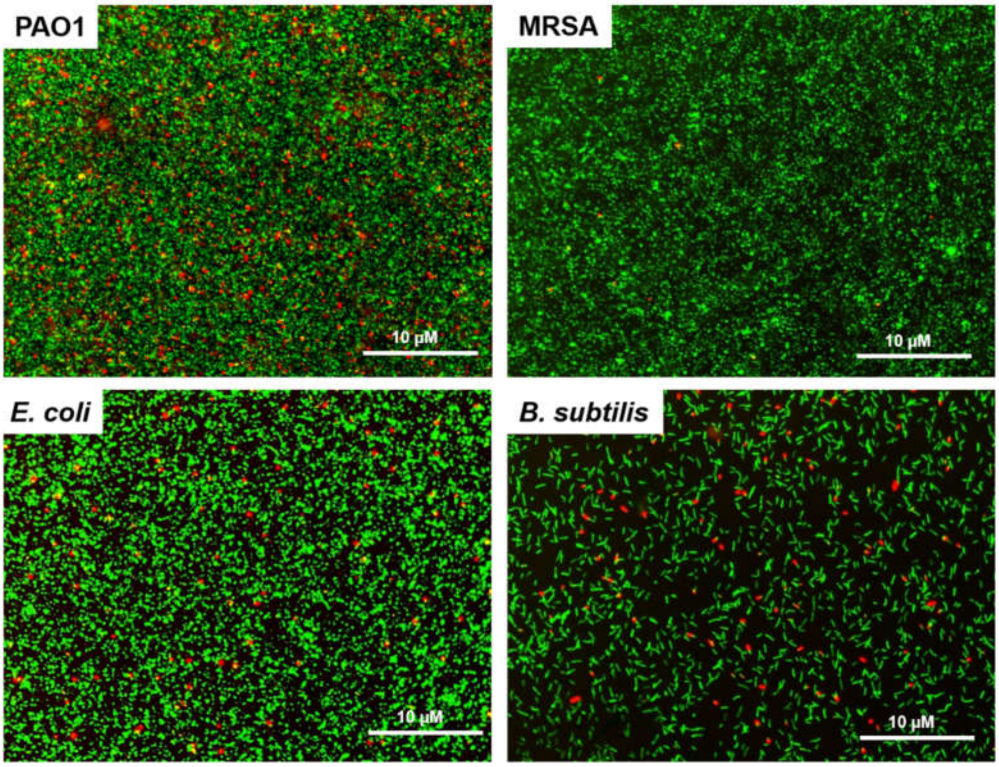


**Figure S6.** Live dead staining of various bacteria on Au-FKC surface.

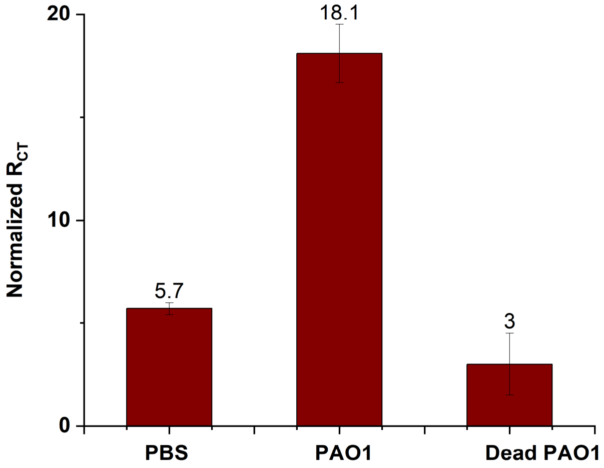


**Figure S7.** EIS measurements of AuE-FKC exposed to dead and live PAO1. PAO1 was killed using addition of 100 µg/mL FKC to bacteria suspension.

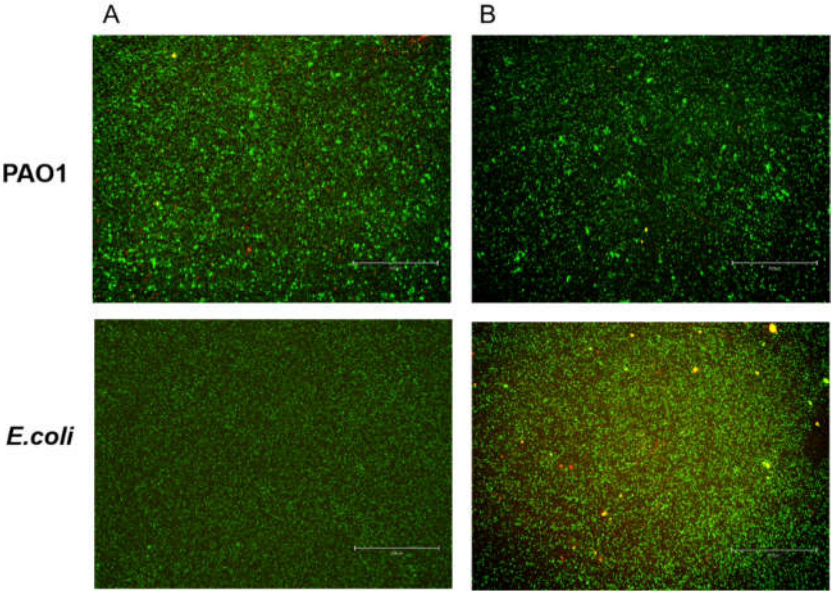


**Figure S8.** Effect in $[Fe(CN)_6]^{3-/4-}$ on the binding of bacteria to FKC surfaces. A. after 10 min incubation with bacteria. B. After additional incubation with $[Fe(CN)_6]^{3-/4-}$. Magnification x10, scale bar – 300 μM.
